# Supplementary material for: Safety and immunogenicity of GamEvac-Combi, a heterologous rVSV- and rAd5-vectored Ebola vaccine: a randomized controlled multicenter clinical trial in the Republic of Guinea and Russia
Source: Front Immunol. 2025 Mar 20;16:1487039. doi: 10.3389/fimmu.2025.1487039 (PMC11979634; doi:10.3389/fimmu.2025.1487039)
Supplement: Supplementary file 1 [file DataSheet1.docx]

Supplementary Material

# Supplementary Authors List – GamEvac-Combi trial group

|  |  |
| --- | --- |
| Konomou Kpakile Victor | Medical service CBK RUSAL, Research Center for Epidemiology, Microbiology and Medical Care, B.P:146, Kindia Republic of Guinea, CREMS (Pastori) |
| Camara Sekou Bakary |  |
| Diallo Mamadou Yero |  |
| Kolie Moussa Claver |  |
| Camara Ibrahima Aissatou |  |
| Soumah Idrissa |  |
| Gbamou N'yereke Jerome |  |
| Shishina Anna Valentinovna | Federal state budgetary military educational institution of higher education «Military Medical Academy named after S.M. Kirov» of the Ministry of Defense of the Russian Federation |
| Beliaevskaia Irina Vladimirovna |  |
| Komissarova Galina Mikhailovna |  |
| Iarotskaia Olga Aleksandrovna |  |
| Glazkova Marina Anatolyevna | National Research Centre of Epidemiology and Microbiology named after Honorary Academician N. F. Gamaleya, Ministry of Health of Russian Federation |
| Telegina Anastasia Aleksandrovna |  |
| Pushkarev Andrey Yurevich |  |

# Supplementary Tables

**Supplementary Table 1.** Systemic and local adverse events by grade, associated with vaccine/placebo administration (AAE). AAEs reported during the whole study are shown in the table as a number of subjects with reported AE and % of the group.

|  | **Grade** | **Vaccine (n = 1904)** | | **Placebo (n = 100)** | | **Overall (n = 2004)** | | **p-value** |
| --- | --- | --- | --- | --- | --- | --- | --- | --- |
| **Overall AEs** | Mild | 532 | 27,94% | 18 | 18,00% | 550 | 27,45% | 0,0299 |
|  | Moderate | 21 | 1,10% | 0 | 0,00% | 21 | 1,05% | 0,2911 |
| **Systemic and local reactions** | **Mild** | **395** | **20,75%** | **13** | **13,00%** | **408** | **20,36%** | **0,0608** |
|  | **Moderate** | **14** | **0,74%** | **0** | **0,00%** | **14** | **0,70%** | **0,3895** |
| Hyperthermia | Mild | 342 | 17,96% | 10 | 10,00% | 352 | 17,56% | 0,0414 |
|  | Moderate | 13 | 0,68% | 0 | 0,00% | 13 | 0,65% | 0,4071 |
| Pain (local) | Mild | 45 | 2,36% | 2 | 2,00% | 47 | 2,35% | 0,8149 |
|  | Moderate | 1 | 0,05% | 0 | 0,00% | 1 | 0,05% | 0,8187 |
| Chills | Mild | 24 | 1,26% | 2 | 2,00% | 26 | 1,30% | 0,5241 |
|  | Moderate | 1 | 0,05% | 0 | 0,00% | 1 | 0,05% | 0,8187 |
| Asthenia | Mild | 19 | 1,00% | 1 | 1,00% | 20 | 1,00% | 0,9984 |
| Malaise | Mild | 6 | 0,32% | 0 | 0,00% | 6 | 0,30% | 0,5740 |
| Erythema (local) | Mild | 2 | 0,11% | 1 | 1,00% | 3 | 0,15% | 0,0241 |
|  | Moderate | 1 | 0,05% | 0 | 0,00% | 1 | 0,05% | 0,8187 |
| Feeling of fever (subjective) | Mild | 1 | 0,05% | 0 | 0,00% | 1 | 0,05% | 0,8187 |
| Fatigue | Mild | 1 | 0,05% | 0 | 0,00% | 1 | 0,05% | 0,8187 |
| **Nervous system** | **Mild** | **216** | **11,34%** | **10** | **10,00%** | **226** | **11,28%** | **0,6786** |
|  | **Moderate** | **3** | **0,16%** | **0** | **0,00%** | **3** | **0,15%** | **0,6912** |
| Headache | Mild | 215 | 11,29% | 10 | 10,00% | 225 | 11,23% | 0,6900 |
|  | Moderate | 3 | 0,16% | 0 | 0,00% | 3 | 0,15% | 0,6912 |
| Dizziness | Mild | 17 | 0,89% | 0 | 0,00% | 17 | 0,85% | 0,3426 |
| **Muscle, skeletal and connective tissue** | **Mild** | **156** | **8,19%** | **4** | **4,00%** | **160** | **7,98%** | **0,1316** |
|  | **Moderate** | **1** | **0,05%** | **0** | **0,00%** | **1** | **0,05%** | **0,8187** |
| Myalgia | Mild | 130 | 6,83% | 3 | 3,00% | 133 | 6,64% | 0,1339 |
|  | Moderate | 1 | 0,05% | 0 | 0,00% | 1 | 0,05% | 0,8187 |
| Arthralgia | Mild | 39 | 2,05% | 3 | 3,00% | 42 | 2,10% | 0,5173 |
| Bone pain | Mild | 2 | 0,11% | 0 | 0,00% | 2 | 0,10% | 0,7457 |
| Back pain | Mild | 1 | 0,05% | 0 | 0,00% | 1 | 0,05% | 0,8187 |
| Musculoskeletal discomfort | Mild | 1 | 0,05% | 0 | 0,00% | 1 | 0,05% | 0,8187 |
| Local (muscle) pain | Mild | 1 | 0,05% | 0 | 0,00% | 1 | 0,05% | 0,8187 |
| **Skin and subcutaneous tissues** | **Mild** | **21** | **1,10%** | **0** | **0,00%** | **21** | **1,05%** | **0,2911** |
|  | **Moderate** | **2** | **0,11%** | **0** | **0,00%** | **2** | **0,10%** | **0,7457** |
| Itch | Mild | 16 | 0,84% | 0 | 0,00% | 16 | 0,80% | 0,3574 |
| Rash | Mild | 4 | 0,21% | 0 | 0,00% | 4 | 0,20% | 0,6464 |
|  | Moderate | 2 | 0,11% | 0 | 0,00% | 2 | 0,10% | 0,7457 |
| Hyperhidrosis | Mild | 2 | 0,11% | 0 | 0,00% | 2 | 0,10% | 0,7457 |
| **Gastrointestinal** | **Mild** | **11** | **0,58%** | **0** | **0,00%** | **11** | **0,55%** | **0,4460** |
| Nausea | Mild | 7 | 0,37% | 0 | 0,00% | 7 | 0,35% | 0,5436 |
| Vomit | Mild | 2 | 0,11% | 0 | 0,00% | 2 | 0,10% | 0,7457 |
| Abdominal pain | Mild | 2 | 0,11% | 0 | 0,00% | 2 | 0,10% | 0,7457 |
| Dyspepsia | Mild | 1 | 0,05% | 0 | 0,00% | 1 | 0,05% | 0,8187 |
| **Infections and infestations** | Mild | 8 | 0,42% | 0 | 0,00% | 8 | 0,40% | 0,5160 |
|  | Moderate | 1 | 0,05% | 0 | 0,00% | 1 | 0,05% | 0,8187 |
| Malaria | Mild | 2 | 0,11% | 0 | 0,00% | 2 | 0,11% | 0,7457 |
|  | Moderate | 1 | 0,05% | 0 | 0,00% | 1 | 0,05% | 0,8187 |
| Gastroenteritis | Mild | 2 | 0,11% | 0 | 0,00% | 2 | 0,10% | 0,7457 |
| Periodontitis | Mild | 1 | 0,05% | 0 | 0,00% | 1 | 0,05% | 0,8187 |
| Pharyngitis | Mild | 1 | 0,05% | 0 | 0,00% | 1 | 0,05% | 0,8187 |
| Typhoid fever | Mild | 1 | 0,05% | 0 | 0,00% | 1 | 0,05% | 0,8187 |
| Nasopharyngitis | Mild | 1 | 0,05% | 0 | 0,00% | 1 | 0,05% | 0,8187 |
| **Cardiac** | **Mild** | **4** | **0,21%** | **0** | **0,00%** | **4** | **0,20%** | **0,6464** |
|  | **Moderate** | **3** | **0,16%** | **0** | **0,00%** | **3** | **0,15%** | **0,6912** |
| Sinus tachycardia | Mild | 1 | 0,05% | 0 | 0,00% | 1 | 0,05% | 0,8187 |
|  | Moderate | 2 | 0,11% | 0 | 0,00% | 2 | 0,10% | 0,7457 |
| Bradycardia | Mild | 1 | 0,05% | 0 | 0,00% | 1 | 0,05% | 0,8187 |
|  | Moderate | 1 | 0,05% | 0 | 0,00% | 1 | 0,05% | 0,8187 |
| Tachycardia | Mild | 1 | 0,05% | 0 | 0,00% | 1 | 0,05% | 0,8187 |
| Heartbeat (subjective palpitation) | Mild | 1 | 0,05% | 0 | 0,00% | 1 | 0,05% | 0,8187 |
| **Metabolic and nutritional** | **Mild** | **5** | **0,26%** | **0** | **0,00%** | **5** | **0,25%** | **0,6079** |
| Loss of appetite | Mild | 4 | 0,21% | 0 | 0,00% | 4 | 0,20% | 0,6464 |
| Appetite disturbance | Mild | 1 | 0,05% | 0 | 0,00% | 1 | 0,05% | 0,8187 |
| **Changes in laboratory variables** | **Mild** | **3** | **0,16%** | **0** | **0,00%** | **3** | **0,15%** | **0,6912** |
|  | **Moderate** | **1** | **0,05%** | **0** | **0,00%** | **1** | **0,05%** | **0,8187** |
| Increased blood pressure | Mild | 3 | 0,16% | 0 | 0,00% | 3 | 0,15% | 0,6912 |
|  | Moderate | 1 | 0,05% | 0 | 0,00% | 1 | 0,05% | 0,8187 |
| **Mental disorders** | **Mild** | **1** | **0,05%** | **0** | **0,00%** | **1** | **0,05%** | **0,8187** |
| Sleep disorder | Mild | 1 | 0,05% | 0 | 0,00% | 1 | 0,05% | 0,8187 |
| **Respiratory** | **Mild** | **1** | **0,05%** | **0** | **0,00%** | **1** | **0,05%** | **0,8187** |
| Rhinorrhea | Mild | 1 | 0,05% | 0 | 0,00% | 1 | 0,05% | 0,8187 |

**Supplementary Table 2.** Death related severe adverse events.

| № | Group | Date of Component B administration | Date of SAE manifestation | Death-related records | Death cause | Causality assessment |
| --- | --- | --- | --- | --- | --- | --- |
| 1 | Vaccine | 10.09.2017 | 19.08.2018 | The volunteer informed the medical staff about experienced severe abdominal pain and was sent for consultation with a surgeon at the regional hospital in Kindia named after Alpha Dialo. Data on fatal outcome was received from the surgeon. | peritonitis | unrelated |
| 2 | Placebo | 11.07.2018 | 17.12.2018 | The volunteer did not attend visit 7 (6 months after first administration). According to contacts (friends and relatives), the volunteer died three weeks ago and did not visit medical institutions | unknown | unrelated |
| 3 | Vaccine | 12.08.2018 | May 2019 | According to contacts (relatives), the volunteer committed suicide in May 2019, exact date is unknown. | suicide | unrelated |
| 4 | Vaccine | 29.06.2018 | Apr 2019 | According to contacts (relatives), the volunteer died in April 2019, after fainted. | unknown | unrelated |
| 5 | Placebo | 16.08.2018 | 22.01.2019 | The volunteer did not attend visit 7 (6 months after first administration). According to contacts (relatives), the volunteer died in June 2019, the death cause was not disclosed to the staff. | unknown | unrelated |
| 6 | Vaccine | 01.09.2018 | 27.05.2019 | The volunteer died due to road accident. | road accident | unrelated |
| 7 | Vaccine | 04.08.2018 | 28.05.2019 | The volunteer did not attend visit 7 (6 months after first administration). Cause of the death remains not clear. | unknown | unrelated |

# Supplementary Figures


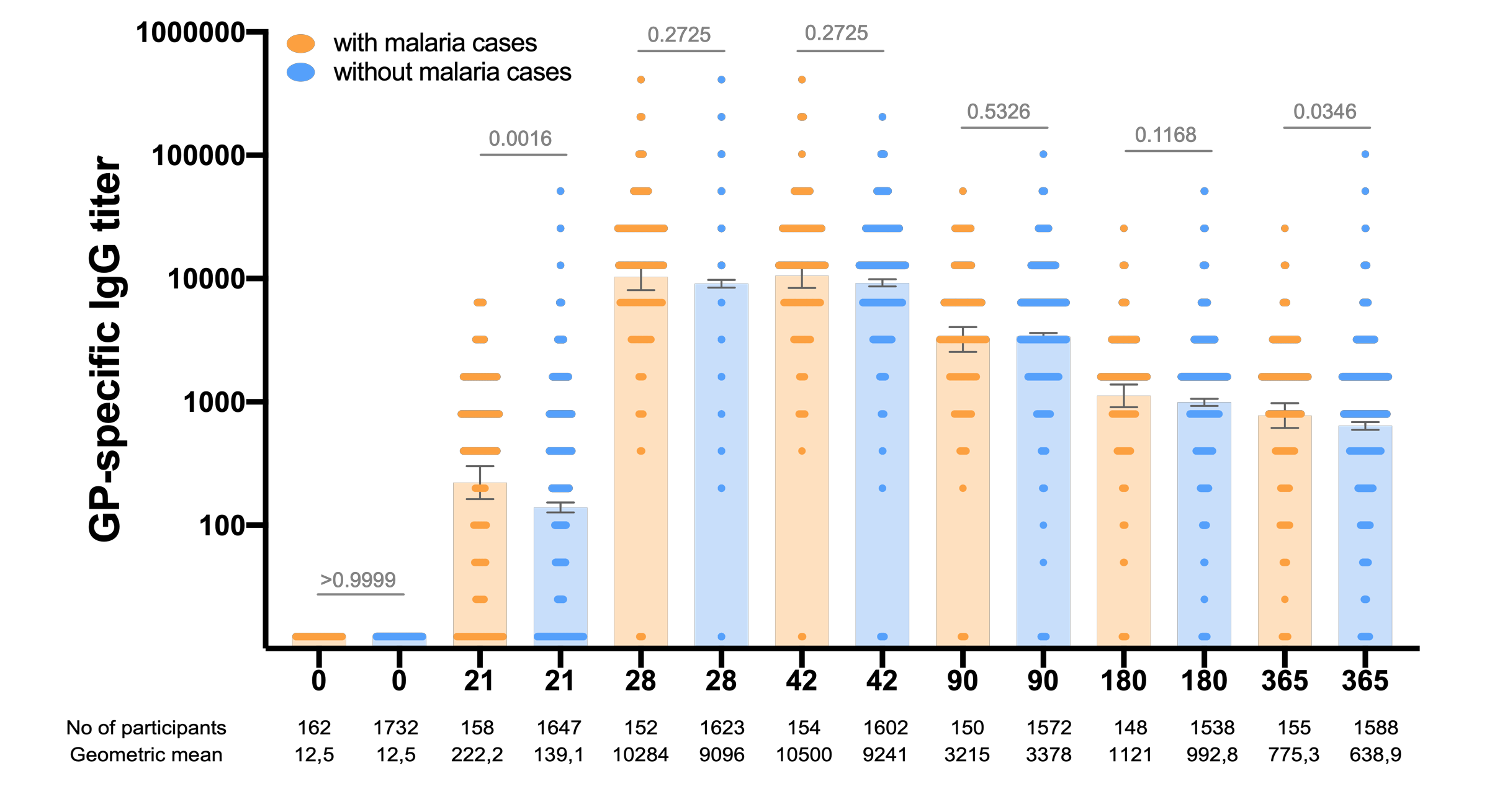


**Supplementary Figure 1.** Humoral immune response in vaccinated participants with and without malaria cases. Humoral immune response in vaccinated participants with and without malaria cases. GP-specific antibody titers at days 0, 21, 28, 42, 90, 180 and 365, as measured by ELISA are shown. Bars show GMT, 95% CI are marked by whiskers. Visits on 3, 6, 12 months are marked as day 90, 180, 365 correspondingly. Differences between IgG titers between groups (convalescents and non-convalescent) were calculated with Mann-Whitney test (p value is marked above grey bars).
